# The Effect of Emotional Valence on Ventricular Repolarization Dynamics Is Mediated by Heart Rate Variability: A Study of QT Variability and Music-Induced Emotions

**DOI:** 10.3389/fphys.2019.01465

**Published:** 2019-11-29

**Authors:** Michele Orini, Faez Al-Amodi, Stefan Koelsch, Raquel Bailón

**Affiliations:** ^1^Institute of Cardiovascular Sciences, University College London, London, United Kingdom; ^2^The William Harvey Research Institute, Queen Mary University of London, London, United Kingdom; ^3^Department of Biological and Medical Psychology, University of Bergen, Bergen, Norway; ^4^Aragon Institute for Engineering Research, University of Zaragoza, Zaragoza, Spain; ^5^Center for Biomedical Research in the Network in Bioengineering, Biomaterials and Nanomedicine (CIBER-BBN), Madrid, Spain

**Keywords:** QT variability, heart rate variability, repolarization, music-induced emotions, emotional valence, time-frequency

## Abstract

**Background:**

Emotions can affect cardiac activity, but their impact on ventricular repolarization variability, an important parameter providing information about cardiac risk and autonomic nervous system activity, is unknown. The beat-to-beat variability of the QT interval (QTV) from the body surface ECG is a non-invasive marker of repolarization variability, which can be decomposed into QTV related to RR variability (QTVrRRV) and QTV unrelated to RRV (QTVuRRV), with the latter thought to be a marker of intrinsic repolarization variability.

**Aim:**

To determine the effect of emotional valence (pleasant and unpleasant) on repolarization variability in healthy volunteers by means of QTV analysis.

**Methods:**

75 individuals (24.5 ± 3.2 years, 36 females) without a history of cardiovascular disease listened to music-excerpts that were either felt as pleasant (*n* = 6) or unpleasant (*n* = 6). Excerpts lasted about 90 s and were presented in a random order along with silent intervals (*n* = 6). QTV and RRV were derived from the ECG and the time-frequency spectrum of RRV, QTV, QTVuRRV and QTVrRRV as well as time-frequency coherence between QTV and RRV were estimated. Analysis was performed in low-frequency (LF), high frequency (HF) and total spectral bands.

**Results:**

The heart rate-corrected QTV showed a small but significant increase from silence (median 347/interquartile range 31 ms) to listening to music felt as unpleasant (351/30 ms) and pleasant (355/32 ms). The dynamic response of QTV to emotional valence showed a transient phase lasting about 20 s after the onset of each musical excerpt. QTV and RRV were highly correlated in both HF and LF (mean coherence ranging 0.76–0.85). QTV and QTVrRRV decreased during listening to music felt as pleasant and unpleasant with respect to silence and further decreased during listening to music felt as pleasant. QTVuRRV was small and not affected by emotional valence.

**Conclusion:**

Emotional valence, as evoked by music, has a small but significant effect on QTV and QTVrRRV, but not on QTVuRRV. This suggests that the interaction between emotional valence and ventricular repolarization variability is mediated by cycle length dynamics and not due to intrinsic repolarization variability.

## Introduction

The beat to beat variability of the QT interval (QTV) of the electrocardiogram is an established measure of ventricular repolarization dynamics and a marker of both cardiovascular risk and cardiac autonomic modulation (Baumert et al., 2016). Since the QTV correlates with the cardiac cycle length through cardiac restitution properties (Orini et al., 2016), QTV is largely affected by RR variability (RRV), which reflects supra-ventricular as opposed to ventricular dynamics. A methodology that separates QTV into two components, one related to RRV and the other unrelated to RRV and thought to represent intrinsic repolarization variability, has been recently proposed (Orini et al., 2018).

Emotions are known to be associated with changes in cardiac function, mediated by the autonomic nervous system. These changes include parameters such as heart rate, heart rate variability and respiration (Steptoe and Brydon, 2009; Steptoe and Kivimäki, 2012). In some studies, emotions have been linked to increased risk of malignant arrhythmias and cardiovascular diseases (Taggart et al., 2011a, b), in particular when related to stress (Steptoe and Kivimäki, 2012). Emotions can be measured along two dimensions: intensity (arousal) and valence (attractiveness versus averseness), with the former exerting a stronger effect on physiological parameters (Hilz et al., 2014). The impact of emotional valence on cardiac repolarization is unknown.

This study investigates for the first time the dynamic interactions between emotional valence and QTV in healthy volunteers, and sought to determine whether the QTV response reflects intrinsic ventricular repolarization dynamics or is mediated by RRV. As in previous studies (Orini et al., 2010; Krabs et al., 2015), emotional states with opposite valence (pleasantness and unpleasantness) were induced by listening to pleasant or noise-like unpleasant music, while silence was used as a baseline control.

## Materials and Methods

### Experimental Set-Up

The experimental set-up was described in details in previous studies (Orini et al., 2010; Krabs et al., 2015) and examples of the acoustic stimuli can be found in Krabs et al. (2015). Briefly, 75 volunteers (age 24.5 ± 3.2 years, 36 female) listened to acoustic stimuli through headphones at a comfortable loudness of around 60 dB in supine position with closed eyes. Participants were exposed to: (1) Six excerpts of pleasant joyful instrumental music (pleasant condition). (2) Six excerpts of isochronous Shepard tones. (3) Six excerpts of isochronous Shepard tones overlaid with unpleasant dissonant music-like noise (unpleasant condition). These were electronically created by recording backward a modified version of the pleasant excerpts, previously simultaneously recorded one semitone above and a tritone below the original pitch. (4) Six intervals of silence (resting condition). All excerpts and intervals of silence lasted about 90 s and were presented in the same randomized order (an example will be described in the “Results” section). All excerpts were matched by tempo and volume in an attempt to control for emotional arousal. Successive excerpts were separated by a 20 s pause during which participants were requested to rate how they felt by pressing response buttons (participants were asked to rate their own emotional state on a six point scale from 1, “very pleasant” to 6, “very unpleasant”). To ensure that participants paid equal attention to all excerpts, they were instructed to listen carefully and to tap the meter of the stimuli with their right index finger. No tapping was required during the resting condition. The study was approved by the ethics committee of the University of Leipzig. Written informed consent was obtained from all participants.

### ECG Analysis

**FIGURE 1:**
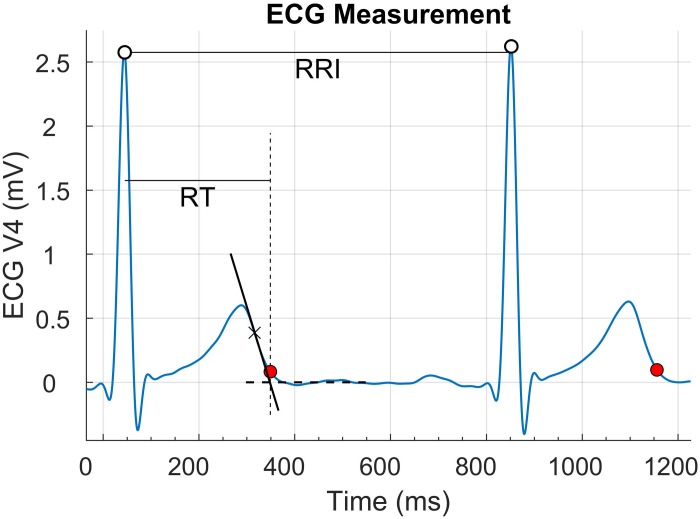
Measurement of RR and QT intervals. White and red circles identify the R-wave and the end of the T-wave, respectively. The RT interval variability was used as robust surrogate for QT variability.

Standard 12 lead electrocardiograms were measured using a 32 MREFA amplifier (Twente Medical Systems, Enschede, Netherlands) and digitized with a sampling rate of 1000 Hz. For the sake of this study, lead V4 was analyzed to derive the main results while lead II was used to assess intra-lead reproducibility. This was chosen because lead V4 usually shows tall R- and T-waves and was therefore assumed to be characterized by high signal-to-noise ratio (Baumert et al., 2016), whereas lead II is one of the most clinically relevant and most widely used lead. The data were analyzed off-line using MATLAB, MathWorks. The peak of the R-wave and the end of the T-wave were detected, with the latter measured using the tangent method (Figure 1). The RT interval was used as a robust estimate of the QTV, which is particularly suitable for analysis of beat to beat variability. In fact, QT and RT variabilities are expected to be very similar, because the beat-to-beat variability of the QRS duration in sinus rhythm in healthy volunteers is negligible, and RT measurement is more robust than QT measurement as the identification of the R-wave peak is easier than the identification of QRS onset. All recordings were revised. Artifacts and ectopic beats were rare and were corrected using a bespoke graphical user interface as in previous studies (Orini et al., 2016, 2017b).

### QTV and HRV Analysis

Time series were evenly resampled at 4 Hz. Time-frequency distributions were used to study the time course of the signals’ spectral components and overcome the limitations of traditional spectral analysis in non-stationary conditions (Cohen, 1989; Hlawatsch and Boudreaux-Bartels, 1992). The Wigner-Ville distribution of RRV and QTV signals were filtered using a kernel designed to provide the minimum amount of time-frequency smoothing while achieving complete elimination of crossterms and time-frequency coherence bounded between zero and one (Orini et al., 2012c, d). Temporal and spectral resolutions were 12.5 s and 0.039 Hz, respectively. The time-frequency coherence between QTV and RRV was computed using previously described algorithms (Orini et al., 2012c). This time-frequency representation provides an assessment of the local linear coupling between the signals’ spectral components in both time and frequency. It ranges from zero to one, and it is equal to one for a given time, **t*_*0*_*, and frequency, *f*_*0*_, if at time *t*_*0*_ the two signals show an oscillation with same instantaneous frequency *f*_*0*_. The time-frequency spectrum of QTV was separated into two components, one representing QTV related to RRV (QTVrRRV) and the other representing QTV unrelated to RRV (QTVuRRV). This was achieved by modulating the time-frequency spectrum of QTV, *S*_*QT*_(*t*,*f*), by the time-frequency coherence between QTV and RRV, γ_*QTV*,*RV*_(*t*,*f*) as demonstrated in Orini et al. (2018):

$$S_{Q\,T\,V\,u\,R\,R}\,\left(t,f\right)=S_{Q\,T\,V}\,\left(t,f\right)-S_{Q\,T\,V\,r\,R\,R\,V}\,\left(t,f\right)=\left(1-{\left|\gamma_{Q\,T\,V,R\,R\,V}\,\left(t,f\right)\right|}^{2}\right)\,S_{Q\,T\,V}\,\left(t,f\right)$$

The time course of the magnitude of low frequency (LF) and high frequency (HF) oscillations were obtained by averaging the time-frequency distributions in the LF (0.04–0.15 Hz) and HF (0.15–0.4) spectral bands. The time course of the total signals’ magnitude was obtained by averaging the time-frequency distributions in the spectral band (0.04–0.4 Hz).

### Statistical Analysis

ECG recordings from five individuals were discarded due to insufficient signal quality.

To assess physiological changes between different conditions, pair-wise comparisons were performed using the Wilcoxon signed-rank test for related samples. 18 physiological indices were considered (see Table 1). For each one of them, the temporal mean was obtained in 50 s long temporal windows starting 20 s after the onset of a given condition to exclude the transient occurring immediately after the condition’s onset (Orini et al., 2010). This provided a single value for each one of the 24 epochs (6 different excerpts × 4 conditions). Values corresponding to the same condition (i.e., pleasant, unpleasant, rest, and Shepard tones) were grouped and averaged to provide a single value per condition per individual. Mathematically, this is described as:

$$X_{i}^{C}=\frac{1}{T\times R}\,\sum_{j=1}^{6}\bar{X_{i}^{C,j}}$$

**TABLE 1:** Median (interquartile range) of cardiac parameters during rest, pleasant, and unpleasant conditions evaluated within the stable phase (20–70 s after the onset of excerpts) are shown on the left.

	**Rest**	**Pleasant**	**Unpleasant**	**Rest vs. pleasant**	**Rest vs. unpleasant**	**Pleasant vs. unpleasant**
RR	878 (163)	835 (182)	864 (179)	**2.11E-10**	**8.00E-10**	**3.62E-04**
QT	333 (37)	328 (36)	329 (35)	**5.30E-08**	**4.31E-09**	9.80E-01
QTc	347 (31)	355 (32)	351 (30)	**9.02E-11**	**8.76E-10**	**5.24E-07**
QTV-LF	0.73 (0.62)	0.56 (0.50)	0.56 (0.47)	**1.28E-05**	6.28E-03	6.28E-03
QTV-HF	0.95 (1.04)	0.67 (0.87)	0.74 (0.89)	**9.27E-08**	**3.80E-06**	**8.92E-05**
QTV-TOT	1.88 (1.72)	1.31 (1.20)	1.45 (1.28)	**2.98E-07**	**2.02E-05**	**1.80E-04**
RRV-LF	577 (583)	289 (371)	363 (357)	**3.99E-08**	**4.36E-06**	**2.22E-05**
RRV-HF	558 (684)	224 (326)	345 (419)	**3.45E-11**	**8.76E-10**	**5.08E-10**
RRV-TOT	1226 (1235)	518 (801)	763 (842)	**2.01E-10**	**1.75E-08**	**1.31E-08**
QTVuRRV-LF	0.11 (0.08)	0.10 (0.10)	0.11 (0.09)	4.78E-02	1.95E-01	9.63E-02
QTVuRRV-HF	0.17 (0.14)	0.17 (0.17)	0.17 (0.17)	3.09E-01	2.70E-01	9.97E-01
QTVuRRV-TOT	0.31 (0.20)	0.28 (0.25)	0.27 (0.25)	7.17E-01	7.11E-01	5.98E-01
QTVrRRV-LF	0.59 (0.50)	0.42 (0.32)	0.48 (0.36)	**9.13E-07**	1.07E-02	3.05E-03
QTVrRRV-HF	0.68 (0.97)	0.46 (0.61)	0.58 (0.67)	**5.08E-10**	**1.13E-07**	**2.50E-06**
QTVrRRV-TOT	1.54 (1.49)	1.04 (0.94)	1.16 (1.11)	**6.35E-09**	**6.35E-06**	**2.22E-05**
Cohe-LF	0.87 (0.10)	0.84 (0.11)	0.85 (0.07)	9.82E-03	4.84E-01	3.82E-02
Cohe-HF	0.80 (0.13)	0.76 (0.17)	0.77 (0.14)	**1.61E-07**	**2.19E-04**	**1.65E-04**
Cohe-TOT	0.81 (0.11)	0.78 (0.14)	0.79 (0.11)	**3.60E-07**	1.07E-03	**1.70E-04**

**P*-values of pairwise tests (signed rank Wilcoxon test) are shown on the right, with *P*-values lower than the Bonferroni-corrected threshold reported in bold. RR: R-R interval; QT: QT interval; QTc: heart rate corrected QT; LF, HF, and TOT: low-frequency, high-frequency, and total spectral bands; QTV and RRV: QT and RR variability, respectively; QTVrRRV and QTVuRRV: QTV related and unrelated to RRV, respectively. Cohe: time-frequency coherence.*

where $X_{i}^{C}$ is a scalar representing a given physiological index *X* for the individual *i* = {1:*N*} during condition *C* = {*Pleasant*,*Unpleasant*,*Rest*,*Shepard*} obtained by averaging the tempotal mean $\bar{X_{i}^{C,j}}$ across epochs *j*.

For the sake of this study, the condition characterized by Shepard tones was not considered and comparisons were performed between pleasant, unpleasant and resting conditions. In total, 54 pairwise tests were performed (5 time-frequency indices × 3 spectral bands × 3 comparisons + 3 time invariant indices × 3 comparisons). Threshold for significance was set at *P* < 9.26 × 10^–4^ after Bonferroni correction.

## Results

There was agreement between the participants’ ratings, with all participants rating the consonant excerpts as more pleasant than the dissonant ones (see Supplementary Figure S1). On a scale from 1 (very pleasant) to 6 (very unpleasant), ratings were equal to (median, 1st–3rd quartiles): 2.4, 1.9–2.9 for silence, 1.8, 1.7–2.2 for pleasant music, 4.7, 4.2–5.2 for noise-like unpleasant music and 4.1, 3.54.5 for Shepard’s tones. All comparisons were highly significant after Bonferroni correction (*P* < 5 × 10^–6^, Wilcoxon signed-rank test).

**FIGURE 2:**
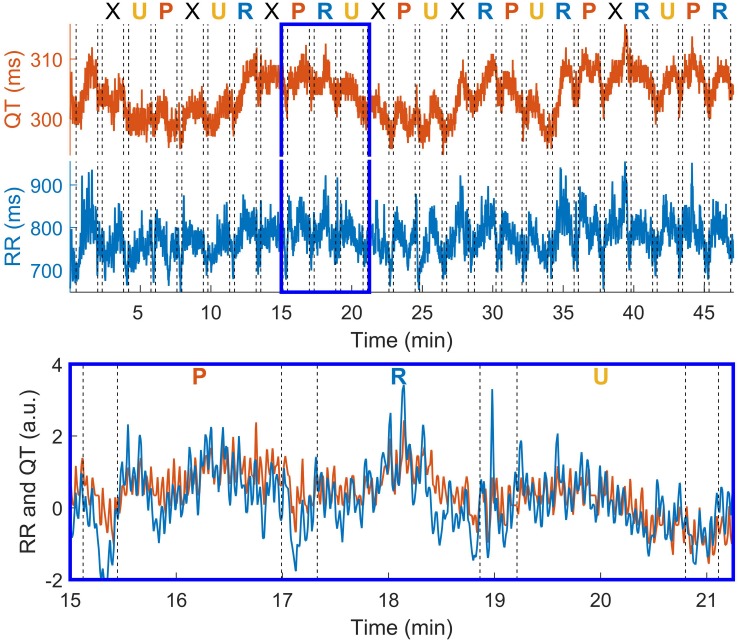
QT and RR interval oscillations in one individual during the entire recording **(top)** and during three consecutive conditions **(bottom)**. Dashed vertical lines represent the beginning and the end of each condition, which are separated by about 20 s pause. The type of condition is reported above the **top panel**. R: rest; P: pleasant music; U: unpleasant music; X: sequence of Shepard tones (not considered in statistical analysis).

A representative example of temporal fluctuations in QT and RR intervals during the entire procedure is shown in Figure 2, where vertical dashed lines represent different epochs. As shown in the inset at the bottom of the figure, QT and RR exhibit similar oscillations and they were therefore characterized by a high level of time-frequency coherence.

Detailed results, including median and interquartile range of all physiological parameters as well as *P*-values for all 54 pair-wise comparisons, are shown in Table 1.

### QT Interval

**FIGURE 3:**
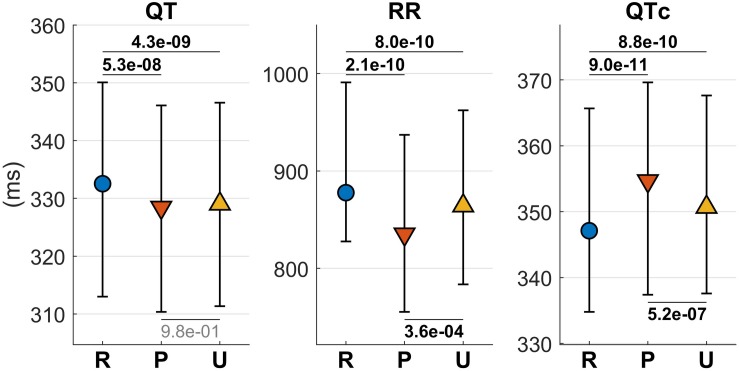
QT, RR, and QTc (QT corrected for heart rate) during listening to pleasant music (P), unpleasant music (U), and rest (R). Markers represent the median values and bars span from the first to the third quartile. *P*-values measuring pair-wise differences are reported in bold if significant and in light gray if not significant.

Changes in the QTV during different conditions are shown in Figure 3. The QTV was significantly shorter during both unpleasant (329/35 ms, median/interquartile range) and pleasant (328/36 ms) than during resting (333/37 ms) condition (*P* < 5.3 × 10^–8^). After correcting for heart rate using the Bazett’s formula, this pattern changed, with corrected QTV increasing from resting (347/41 ms) to unpleasant (351/30 ms) to pleasant (355/32 ms) (*P* < 5 × 10^–7^) (Figure 3).

### QTV Related and Unrelated to RRV

**FIGURE 4:**
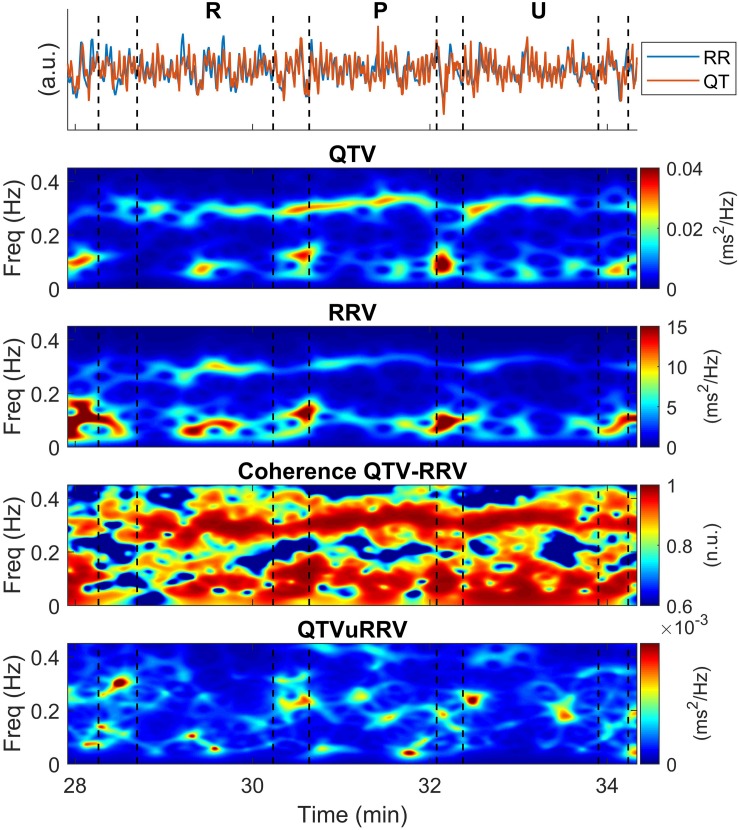
Example of time-frequency representations in a representative individual. The same interval including three consecutive epochs (resting, pleasant and unpleasant conditions) shown in Figure 2 is analyzed. From **top** to **bottom**: QTV and RRV superimposed and normalized to show same amplitude, QTV and RRV time-frequency spectra, time-frequency coherence between QTV and RRV and time-frequency spectrum of QTV unrelated to RRV. R: Rest; P: Pleasant condition; U: Unpleasant condition.

A representative example of time-frequency representations during three consecutive epochs (resting, pleasant and unpleasant) is shown in Figure 4. These include the time-frequency spectra of QTV and RRV, *S*_*QT*_(*t*,*f*) and *S*_*RRV*_(*t*,*f*) respectively, the time-frequency coherence between QTV and RRV, γ_*QTV*,*RV*_(*t*,*f*)and the time-frequency spectrum of QTVuRRV, *S*_*QTVuRRV*_(*t*,*f*) Changes in the patterns of color in these time-frequency representations indicate changes in the magnitude and frequency of the signals’ spectral components typical of non-stationary conditions. QTV and RRV showed similar time-frequency structures and high coherence. This implies that most of the spectral content of QTV was related to RRV and QTVuRRV was much smaller than QTV (note the different scale of the color bars in Figure 4).

**FIGURE 5:**
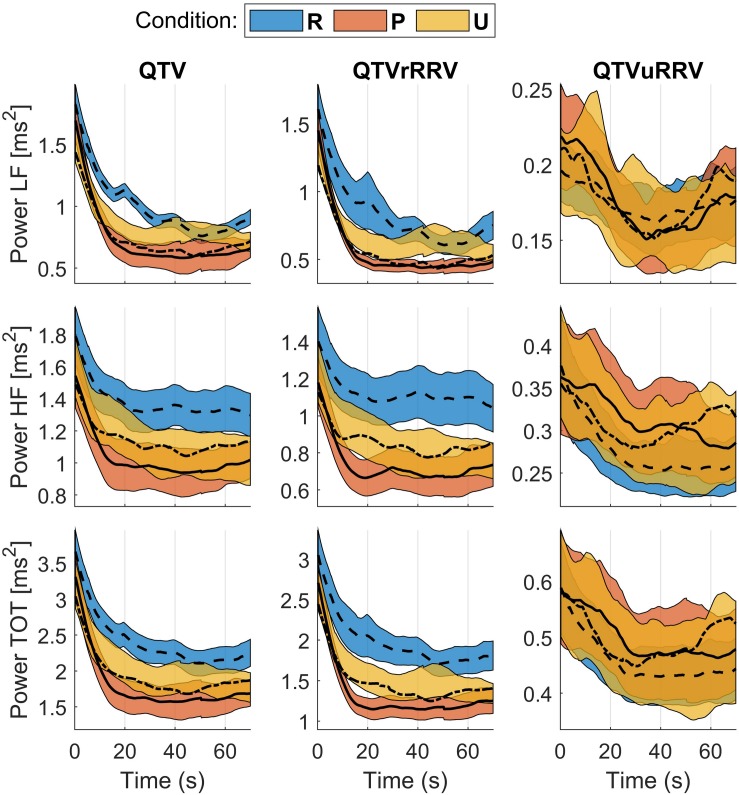
QTV trends. From **left** to **right**: QTV, QTV related to RRV (QTVrRRV), and QTV unrelated to RRV (QTVrRRV). From **top** to **bottom**: instantaneous power of LF, HF, and total spectral band. Solid lines represent the mean trend across all individuals and shaded areas represent standard error. R: rest; P: pleasant condition; U: unpleasant condition.

The time course of the QTV’s spectral components (instantaneous power) presented two phases (Figure 5): A sharp decrease with respect to the preceding interval (the pause between two consecutive epochs during which the individuals were asked to rate how they felt) during the first 20 s with subsequent stabilization during the remaining 50–60 s until the end of the epoch. QTVrRRV showed the biggest intra-condition changes (Figure 5, middle panel) whereas QTVuRRV showed little intra-condition changes as demonstrated by overlapping trends in the right hand side of Figure 5.

**FIGURE 6:**
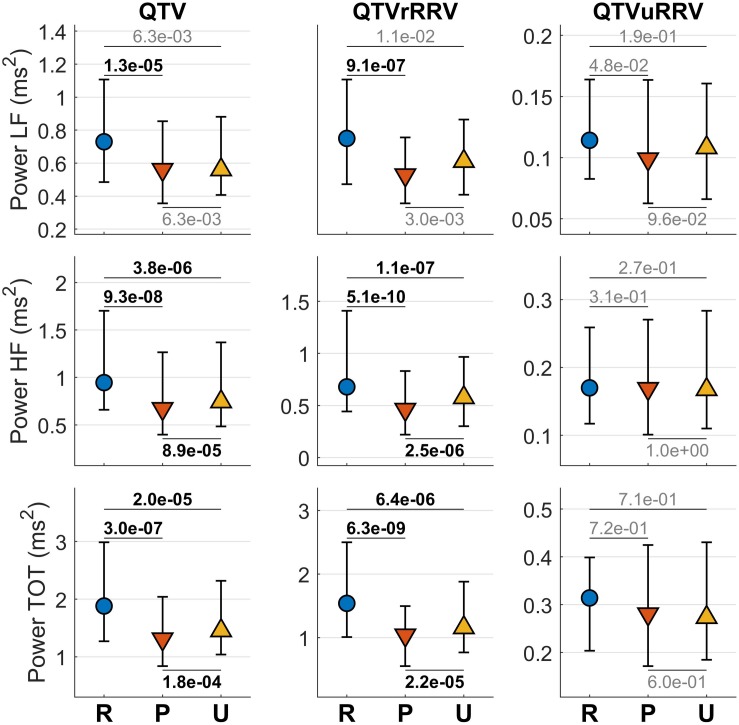
QTV and QTV related and unrelated to RRV during listening to pleasant music (P), unpleasant music (U), and rest (R). From **left** to **right**: QTV, QTV related to RRV (QTVrRRV), and QTV unrelated to RRV (QTVrRRV). From **top** to **bottom**: mean power of LF, HF and total spectral band. Markers represent the median values and bars span from the first to the third quartile. *P*-values measuring pair-wise differences are reported in bold if significant and in light gray if not significant.

Figure 6 shows the distribution of mean QTV, QTV related and unrelated to RRV during the stable phase of the recordings (from 20 to 50 s after the onset of each epoch). QTV and QTVrRRV show similar patterns, with oscillations of higher magnitude in all spectral band during rest than during pleasant and unpleasant conditions, and with lower magnitude for HF oscillations and total power during pleasant than unpleasant condition. The time-frequency coherence between QTV and RRV was high (0.76–0.85) in all spectral bands for all conditions. In HF, a small but significant decrease in coherence was observed from rest (0.80/0.13) to unpleasant (0.77/0.14) to pleasant (0.76/0.17) conditions (Table 1). QTVuRRV was much smaller than QTVrRRV and did not show any significant change in any spectral band (Table 1).

### Intra-Lead Reproducibility

The entire analysis was repeated using beat to beat QTVs obtained from lead II for assessment of intra-lead reproducibility. Correlation between the time series of QTVs from lead V4 and lead II was high, with Spearman’s correlation coefficient equal to 0.95/0.09. However, the correlation between QTV was lower at 0.59/0.26. A lower intra-lead correlation for QTV than for the QTV is expected as QTV has a much lower magnitude than the QTV series, which show very slow oscillations (i.e., very low frequency components with frequency <0.03 Hz) that are removed from QTV. The standard deviation of the QTV signals (entire recording) across all patients was slightly higher in lead II than V4 at 1.8/0.7 ms versus 1.7/0.6 ms, *P* < 0.001, and their correlation was equal to *cc* = 0.78. There was no difference in the SNR of the leads V4 and II (*P* = 0.60). The correlation coefficient between the standard deviation of QTV and SNR was equal to −0.49 (*P* = 8.1 × 10^–5^) in lead V4 and −0.60 in lead II (*P* = 3.6 × 10^–7^). This suggests that QTV in lead V4 was less affected by noise than QTV in lead II.

During the different conditions (silence, pleasant and unpleasant), changes in the QTV and QTc from lead II (Supplementary Figure S2) mirrored those from lead V4. Changes in QTV, QTVrRRV, and QTVuRRV followed a similar pattern in both leads. However, in lead II some differences were no longer significant after Bonferroni’s correction (Supplementary Figure S3).

## Discussion

This study investigated the effect of emotional valence on cardiac repolarization and repolarization dynamics by analyzing the QTV response to music. The main findings are: (1) The QTV decreases during both unpleasant and pleasant emotional states, mirroring similar changes in the RR interval. This pattern is reversed after correction for heart rate, with QTc showing small but significant increase during listening to both pleasant and unpleasant music compared to silence, and during pleasant compared to unpleasant music. (2) The dynamic response of QTV to emotional valence showed a transient phase of about 20 s. (3) Because of the strong coupling between QTV and RRV, both QTV and QTVrRRV followed a similar pattern showing a decrease in variability during both pleasant and unpleasant conditions with respect to the resting condition and a further decrease during pleasant condition with respect to unpleasant condition in HF and in the total spectral components. (4) QTVuRRV was small and not affected by emotional valence.

The existence of a link between emotions or psychological stress and cardiovascular mortality has been demonstrated by many studies (Steptoe and Brydon, 2009). Strong emotions, i.e., characterized by a high level of arousal, have an acute impact on the cardiovascular function and can serve as triggers for arrhythmias and cardiovascular disease, mainly through a complex interaction with the autonomic nervous system (Kreibig, 2010; Taggart et al., 2011b). While it is accepted that emotional arousal (activating versus deactivating) has a stronger effect on human physiology than emotional valence (positive versus negative feeling), the latter has been less investigated and its effect on cardiac repolarization remained undetermined. This is the first study to investigate the simultaneous interaction between emotional valence and QT dynamics. A unique feature of this study is that the experimental set-up was designed to control for arousal by matching excerpts by tempo and volume with the intent of focusing on the effect of felt pleasantness with respect to unpleasantness.

Previous studies have demonstrated a link between intense emotions and potentially pro-arrhythmic repolarization changes (Ziegelstein, 2007; Lampert, 2016). In patients with a history of ventricular arrhythmia, psychological stress induces autonomically mediated repolarization changes (Lampert et al., 2005), while anger-induced T-wave alternans, a marker of repolarization variability (Orini et al., 2019), predicts future ventricular arrhythmias (Lampert et al., 2009). In patients with structurally normal hearts, mental stress altered repolarization inhomogeneity balance (Taggart et al., 2005) and dispersion of repolarization (Child et al., 2014; Finlay et al., 2016). A recent study has shown that not only acute and strong emotions, but also subtle everyday fluctuations in emotional arousal can affect repolarization, with a more noticeable impact in patients with Long QT syndrome and ischemic heart disease (Lane et al., 2018). In the same study, similar QT changes were observed as a response of emotions characterized by both positive and negative valence, which may suggest that the effect of everyday emotions could be primarily a function of arousal.

Music-induced emotions affect several cerebrovascular and cardiovascular parameters, especially heart rate and heart rate variability (Koelsch and Jancke, 2015). Although music-induced emotions may have a smaller impact on cardiac activity as compared to other types of emotions, evidence shows that music can reduce pain and anxiety, and that relaxing music is associated with lower heart rate and blood pressure (Koelsch and Jancke, 2015), which could be beneficial in particular in patients with cardiovascular disease.

The interest in the QTV response to music-induced emotions is motivated by the fact that QTV, and in particular QTVuRRV, provides an indirect assessment of ventricular repolarization variability, which is believed to be modulated by sympathetic drive directed to the ventricles (Porta et al., 2010; El-Hamad et al., 2015). Recent studies have demonstrated the existence of respiratory and LF oscillations in the ventricular action potential of the intact human heart during steady state ventricular pacing and therefore unrelated to cycle length variations (Hanson et al., 2014; Van Duijvenboden et al., 2016; Porter et al., 2019). Several studies have shown that indices of ventricular repolarization, mainly based on QTV, are associated with cardiac risk (Tereshchenko et al., 2009; Oosterhoff et al., 2011; Baumert et al., 2016), with periodic repolarization dynamics being affected by music (Cerruto et al., 2017). The mechanisms promoting intrinsic ventricular repolarization variability are still under investigation, but may imply both the autonomic nervous system (adrenergic stimulation) and mechano-electric feedback (Pueyo et al., 2016; Orini et al., 2017a).

One of the main results of this study is that in young healthy individuals listening to pleasant and unpleasant music, QTV dynamics are largely determined by RRV, whereas QTVuRRV remains stable. This highlights the importance of separating RRV-related and unrelated components to reveal intrinsic repolarization variability (Orini et al., 2018).

In this cohort of young healthy volunteers, the effect of emotional valence on QT and QTV was significant but relatively small, whereas its effect on QTVuRRV was not significant. Although these findings may not have an immediate impact on clinical practice, they provide valuable information to advance our understanding of the interplay between emotions and cardiac disease. Further research is needed to test the effect of emotional valence in the context of preexisting cardiac disorders and to better understand how to translate these findings in strategies that can impact patients’ health. The observation that valance and not only arousal affects the QTV is interesting, because it suggests that potentially clinically relevant changes in arrhythmogenic substrates may be triggered by emotions unrelated to dramatic events. For instance, a small but significant increase in QTc associated with pleasant emotions may be relevant for arrhythmogenesis in the context of repolarization disorders such as long QT syndrome. A recent study analyzing the effect of everyday emotions on the QTV has suggested that in patients with heart conditions (long QT syndrome and ischemic heart disease), but not in healthy individuals, these can affect arrhythmia susceptibility (Lane et al., 2018). Interestingly, although the authors concluded that emotional arousal had a predominant effect with respect to valance, they observed a prolongation of QTc with positive and low-arousal emotions, which, despite important methodological differences between the two studies, is in agreement with our findings. Of note, silence has been previously reported to have a strong relaxing effect on the respiratory rate, heart rate and blood pressure (Bernardi et al., 2006; Orini et al., 2010). The finding that QTc decreases while QTV increases during silence provides further support to the protective effect of relaxation that could be used in specific cohorts to reduce cardiovascular risk (Schneider et al., 2005).

### Limitations and Future Directions

Although this study is based on a relatively large cohort, it only includes young healthy individuals. The presentence of heart disease may amplify the effect of emotions (Lampert, 2016; Lane et al., 2018) and future studies should include patients with a pre-existing arrhythmogenic substrate. Emotions were induced using musical excerpts. Functional neuroimaging studies using similar stimuli have demonstrated that music can modulate activity in brain structures that are known to be crucially involved in emotion (Koelsch et al., 2006; Koelsch, 2014). Future studies are needed to determine if the effect of music-induced emotions can be generalized to other types of emotions and psychological stress. Inter-subject variability in the emotional predisposition to and elaboration of the stimuli were not controlled during the study and may have played a role in the physiological response. For instance, interoceptive awareness has been shown to modulate the heart rate response to emotional pictures (Pollatos et al., 2007) and may also play a role in the modulation cardiac repolarization. This may be assessed in future studies. Although the tangent method is a standard method for identifying the end of the T-wave, it has some limitations (Baumert et al., 2016). Although results obtained from the analysis of lead V4 (main text) generally correlated with those obtained from lead II (Supplementary Material), the statistical significance of some differences differ, especially in the LF band of QTV. Intra-lead differences in QTV have been previously reported and linked to T-wave amplitude, with QTV increasing in leads showing smaller T-waves (Hasan et al., 2012). This is in agreement with our finding that QTV in lead V4, which shows taller T-waves than lead II, was less affected by noise than QTV in lead II. Thus, results obtained from the analysis of V4 are more robust. Although these intra-lead differences are partially due to the technical challenge of measuring small amplitude oscillations of the order of few ms, ECG leads capture repolarization dynamics of different cardiac segments (Srinivasan et al., 2019) and small intra-leads differences may also partially reflect a spatially heterogeneous response of ventricular repolarization.

Finally, although the time-frequency approach implemented in this study is built upon a robust framework that has been tested in several studies (Orini et al., 2012a,b,c,d, 2018), other approaches to decompose QT variability in its different component exist (Porta et al., 2010, 2017; El-Hamad et al., 2015) and future studies may investigate the reproducibility of these findings.

## Conclusion

Emotional valence, as evoked by music, has a small but significant effect on beat-to-beat repolarization variability and this effect is principally mediated by heart rate variability.

## Data Availability Statement

All datasets generated for this study are included in the article/Supplementary Material.

## Ethics Statement

The studies involving human participants were reviewed and approved by the Max Plank Institute, Leipzig, Germany. The patients/participants provided their written informed consent to participate in this study.

## Author Contributions

MO contributed to the design of the analysis. MO and FA-A contributed to the data and statistical analysis. MO and RB contributed to the methodological development. SK contributed to the experimental set-up. MO contributed to the drafting of the work. MO, FA-A, SK, and RB contributed to the critical revision and proofreading.

## Conflict of Interest

The authors declare that the research was conducted in the absence of any commercial or financial relationships that could be construed as a potential conflict of interest.

## References

[B1] Baumert M. Porta A. Vos M. A. Malik M. Couderc J. P. Laguna P. (2016). QT interval variability in body surface ECG: measurement, physiological basis, and clinical value: position statement and consensus guidance endorsed by the European Heart Rhythm Association jointly with the ESC Working Group on cardiac cellular electroph. *Europace* 18 925–944. 10.1093/europace/euv405 26823389 PMC4905605

[B2] Bernardi L. Porta C. Sleight P. (2006). Cardiovascular, cerebrovascular, and respiratory changes induced by different types of music in musicians and non-musicians: the importance of silence. *Heart* 92 445–452. 10.1136/hrt.2005.064600 16199412 PMC1860846

[B3] Cerruto G. Mainardi L. Koelsch S. Orini M. (2017). The periodic repolarization dynamics index identifies changes in ventricular repolarization oscillations associated with musicinduced emotions. *Comput. Cardiol.* 44 2–5. 10.22489/CinC.2017.259-372

[B4] Child N. Hanson B. Bishop M. Rinaldi C. A. Bostock J. Western D. (2014). Effect of mental challenge induced by movie clips on action potential duration in normal human subjects independent of heart rate. *Circ. Arrhythmia Electrophysiol.* 7 518–523. 10.1161/CIRCEP.113.000909 24833641 PMC4143747

[B5] Cohen L. (1989). Time-frequency distributions-a review. *Proc. IEEE* 77 941–981. 10.1109/5.30749

[B6] El-Hamad F. Lambert E. Abbott D. Baumert M. (2015). Relation between QT interval variability and muscle sympathetic nerve activity in normal subjects. *Am. J. Physiol. Hear. Circ. Physiol.* 309 H1218–H1224. 10.1152/ajpheart.00230.2015 26276814

[B7] Finlay M. C. Lambiase P. D. Ben-Simon R. Taggart P. (2016). Effect of mental stress on dynamic electrophysiological properties of the endocardium and epicardium in humans. *Heart Rhythm* 13 175–182. 10.1016/j.hrthm.2015.08.011 26272521 PMC4703839

[B8] Hanson B. Child N. Van Duijvenboden S. Orini M. Chen Z. Coronel R. (2014). Oscillatory behavior of ventricular action potential duration in heart failure patients at respiratory rate and low frequency. *Front. Physiol.* 5:414 10.3389/fphys.2014.00414 PMC4211392 25389408

[B9] Hasan M. A. Abbott D. Baumert M. (2012). Relation between beat-to-beat QT interval variability and t-wave amplitude in healthy subjects. *Ann. Noninvasive Electrocardiol.* 17 195–203. 10.1111/j.1542-474X.2012.00508.x 22816538 PMC6932369

[B10] Hilz M. J. Stadler P. Gryc T. Nath J. Habib-Romstoeck L. Stemper B. (2014). Music induces different cardiac autonomic arousal effects in young and older persons. *Auton. Neurosci. Basic Clin.* 183 83–93. 10.1016/j.autneu.2014.02.004 24636674

[B11] Hlawatsch F. Boudreaux-Bartels G. F. F. (1992). Linear and quadratic time-frequency signal representations. *IEEE Signal. Process. Mag.* 9 21–67. 10.1109/79.127284

[B12] Koelsch S. (2014). Brain correlates of music-evoked emotions. *Nat. Rev. Neurosci.* 15 170–180. 10.1038/nrn3666 24552785

[B13] Koelsch S. Fritz T. Cramon D. Y. V. Müller K. Friederici A. D. (2006). Investigating emotion with music: an fMRI study. *Hum. Brain Mapp.* 27 239–250. 10.1002/hbm.20180 16078183 PMC6871371

[B14] Koelsch S. Jancke L. (2015). Music and the heart. *Eur. Heart J.* 36 3043–3048. 10.1093/eurheartj/ehv430 26354957

[B15] Krabs R. U. Enk R. Teich N. Koelsch S. (2015). Autonomic effects of music in health and Crohn’s disease: the impact of isochronicity, emotional valence, and tempo. *PLoS One* 10:e0126224. 10.1371/journal.pone.0126224 25955253 PMC4425535

[B16] Kreibig S. D. (2010). Autonomic nervous system activity in emotion: a review. *Biol. Psychol.* 84 394–421. 10.1016/j.biopsycho.2010.03.010 20371374

[B17] Lampert R. (2016). Mental stress and ventricular arrhythmias. *Curr. Cardiol. Rep.* 18:118. 10.1007/s11886-016-0798-796 27796855

[B18] Lampert R. Shusterman V. Burg M. McPherson C. Batsford W. Goldberg A. (2009). Anger-induced T-Wave alternans predicts future ventricular arrhythmias in patients with implantable cardioverter-defibrillators. *J. Am. Coll. Cardiol.* 53 774–778. 10.1016/j.jacc.2008.10.053 19245968 PMC3979284

[B19] Lampert R. Shusterman V. Burg M. M. Lee F. A. Earley C. Goldberg A. (2005). Effects of psychologic stress on repolarization and relationship to autonomic and hemodynamic factors. *J. Cardiovasc. Electrophysiol.* 16 372–377. 10.1046/j.1540-8167.2005.40580.x 15828878

[B20] Lane R. D. Reis H. T. Hsu C.-H. Kern K. B. Couderc J. P. Moss A. J. (2018). Abnormal repolarization duration during everyday emotional arousal in long QT syndrome and Coronary Artery disease. *Am. J. Med.* 131:565-572.e2. 10.1016/j.amjmed.2017.12.017 29309742 PMC9423041

[B21] Oosterhoff P. Tereshchenko L. G. Van Der Heyden M. A. G. Ghanem R. N. Fetics B. J. Berger R. D. (2011). Short-term variability of repolarization predicts ventricular tachycardia and sudden cardiac death in patients with structural heart disease: a comparison with QT variability index. *Heart Rhythm* 8 1584–1590. 10.1016/j.hrthm.2011.04.033 21699842

[B22] Orini M. Bailón R. Enk R. Koelsch S. Mainardi L. Laguna P. (2010). A method for continuously assessing the autonomic response to music-induced emotions through HRV analysis. *Med. Biol. Eng. Comput.* 48 423–433. 10.1007/s11517-010-0592-3 20300873

[B23] Orini M. Bailón R. Laguna P. Mainardi L. T. Barbieri R. (2012a). A multivariate timefrequency method to characterize the influence of respiration over heart period and arterial pressure. *EURASIP J. Adv. Signal Process.* 2012:214 10.1186/1687-6180-2012-2214

[B24] Orini M. Bailón R. Mainardi L. Laguna P. (2012b). Synthesis of HRV signals characterized by predetermined time-frequency structure by means of time-varying ARMA models. *Biomed. Signal. Process. Control* 7 141–150. 10.1016/j.bspc.2011.05.003

[B25] Orini M. Bailon R. Mainardi L. T. Laguna P. Flandrin P. (2012c). Characterization of dynamic interactions between cardiovascular signals by time-frequency coherence. *IEEE Trans. Biomed. Eng.* 59 663–673. 10.1109/TBME.2011.2171959 22155936

[B26] Orini M. Laguna P. Mainardi L. T. T. Bailón R. (2012d). Assessment of the dynamic interactions between heart rate and arterial pressure by the cross time–frequency analysis. *Physiol. Meas.* 33 315–331. 10.1088/0967-3334/33/3/315 22354110

[B27] Orini M. Nanda A. Yates M. Di Salvo C. Roberts N. Lambiase P. D. (2017a). Mechanoelectrical feedback in the clinical setting: current perspectives. *Prog. Biophys. Mol. Biol.* 130(Pt B), 365–375. 10.1016/j.pbiomolbio.2017.06.001 28587763

[B28] Orini M. Tinker A. Munroe P. B. Lambiase P. D. (2017b). Long-term intra-individual reproducibility of heart rate dynamics during exercise and recovery in the UK Biobank cohort. *PLoS One* 12:e0183732. 10.1371/journal.pone.0183732 28873397 PMC5584807

[B29] Orini M. Pueyo E. Laguna P. Bailon R. (2018). A time-varying nonparametric methodology for assessing changes in QT variability unrelated to heart rate variability. *IEEE Trans. Biomed. Eng.* 65 1443–1451. 10.1109/TBME.2017.2758925 28991727

[B30] Orini M. Taggart P. Srinivasan N. Hayward M. Lambiase P. D. (2016). Interactions between activation and repolarization restitution properties in the intact human heart: in-vivo whole-heart data and mathematical description. *PLoS One* 11:e0161765. 10.1371/journal.pone.0161765 27588688 PMC5010207

[B31] Orini M. Yanni J. Taggart P. Hanson B. Hayward M. Smith A. (2019). Mechanistic insights from targeted molecular profiling of repolarization alternans in the intact human heart. *Europace* 21 981–989. 10.1093/europace/euz007 30753421 PMC6545501

[B32] Pollatos O. Herbert B. M. Matthias E. Schandry R. (2007). Heart rate response after emotional picture presentation is modulated by interoceptive awareness. *Int. J. Psychophysiol.* 63 117–124. 10.1016/j.ijpsycho.2006.09.003 17137662

[B33] Porta A. Bari V. De Maria B. Baumert M. (2017). A network physiology approach to the assessment of the link between sinoatrial and ventricular cardiac controls. *Physiol. Meas.* 38 1472–1489. 10.1088/1361-6579/aa6e95 28430108

[B34] Porta A. Tobaldini E. Gnecchi-Ruscone T. Montano N. (2010). RT variability unrelated to heart period and respiration progressively increases during graded head-up tilt. *Am. J. Physiol. Heart Circ. Physiol.* 298 H1406–H1414. 10.1152/ajpheart.01206.2009 20154259

[B35] Porter B. Bishop M. J. Claridge S. Child N. Van Duijvenboden S. Bostock J. (2019). Left ventricular activation-recovery interval variability predicts spontaneous ventricular tachyarrhythmia in patients with heart failure. *Heart Rhythm* 16 702–709. 10.1016/j.hrthm.2018.11.013 30528448

[B36] Pueyo E. Orini M. Rodríguez J. F. J. F. Taggart P. (2016). Interactive effect of betaadrenergic stimulation and mechanical stretch on low-frequency oscillations of ventricular action potential duration in humans. *J. Mol. Cell. Cardiol.* 97 93–105. 10.1016/j.yjmcc.2016.05.003 27178727

[B37] Schneider R. H. Alexander C. N. Staggers F. Rainforth M. Salerno J. W. Hartz A. (2005). Long-term effects of stress reduction on mortality in persons = 55 years of age with systemic hypertension. *Am. J. Cardiol.* 95 1060–1064. 10.1016/j.amjcard.2004.12.058 15842971 PMC1482831

[B38] Srinivasan N. T. Orini M. Providencia R. Simon R. Lowe M. Segal O. R. (2019). Differences in the upslope of the precordial body surface ECG T wave reflect right to left dispersion of repolarization in the intact human heart. *Heart Rhythm* 16 943–951. 10.1016/j.hrthm.2018.12.006 30550836 PMC6546969

[B39] Steptoe A. Brydon L. (2009). Emotional triggering of cardiac events. *Neurosci. Biobehav. Rev.* 33 63–70. 10.1016/j.neubiorev.2008.04.010 18534677

[B40] Steptoe A. Kivimäki M. (2012). Stress and cardiovascular disease. *Nat. Rev. Cardiol.* 9 360–370. 10.1038/nrcardio.2012.45 22473079

[B41] Taggart P. Boyett M. R. Logantha S. J. R. J. Lambaise P. D. (2011a). Anger, emotion, and arrythimias: from brain to heart. *Front Physiol.* 2:67 10.3389/fphys.2011.00067 PMC3196868 22022314

[B42] Taggart P. Critchley H. Lambiase P. D. (2011b). Heart-brain interactions in cardiac arrhythmia. *Heart* 97 698–708. 10.1136/hrt.2010.209304 21367742

[B43] Taggart P. Sutton P. Redfern C. Batchvarov V. N. Hnatkova K. Malik M. (2005). The effect of mental stress on the non-dipolar components of the T wave: modulation by hypnosis. *Psychosom. Med.* 67 376–383. 10.1097/01.psy.0000160463.10583.88 15911899

[B44] Tereshchenko L. G. Fetics B. J. Domitrovich P. P. Lindsay B. D. Berger R. D. (2009). Prediction of ventricular tachyarrhythmias by intracardiac repolarization variability analysis. *Circ. Arrhythmia Electrophysiol.* 2 276–284. 10.1161/CIRCEP.108.829440 19808478

[B45] Van Duijvenboden S. Orini M. Child N. Gill J. S. Taggart P. Hanson B. (2016). Investigation of causal interactions between ventricular action potential duration, blood pressure and respiration. *Comput. Cardiol.* 42 621–624. 10.1109/CIC.2015.7410987

[B46] Ziegelstein R. C. (2007). Acute emotional stress and cardiac arrhythmias. *J. Am. Med. Assoc.* 298 324–329. 10.1001/jama.298.3.324 17635893

