# Supplementary material for: The Effect of Emotional Valence on Ventricular Repolarization Dynamics Is Mediated by Heart Rate Variability: A Study of QT Variability and Music-Induced Emotions
Source: Front Physiol. 2019 Nov 29;10:1465. doi: 10.3389/fphys.2019.01465 (PMC6895139; doi:10.3389/fphys.2019.01465)
Supplement: Supplementary file 1 [file Data_Sheet_1.PDF]

Supplementary Material

1 Supplementary Figures

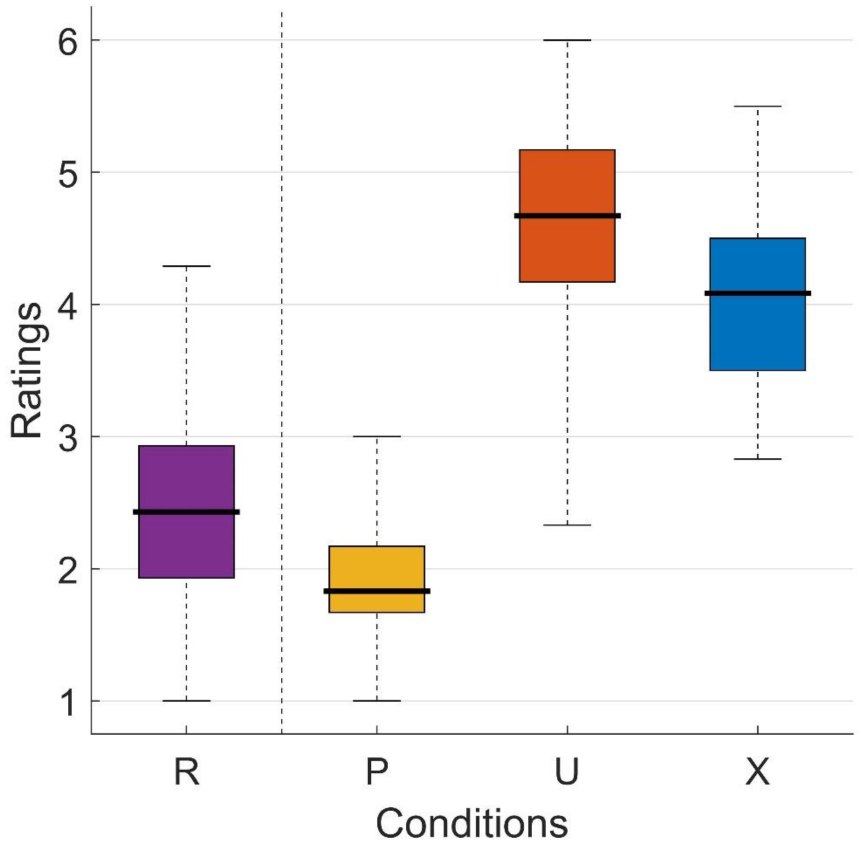

Supplementary Figure 1. Participants' ratings. Boxplots show median (black horizontal line), interquartile range (box) and range (whiskers) for ratings for all participants (n=75) during the listening of pleasant music (P), unpleasant noise-like sounds (U), Shepard's tone (X) and during silence (R). The scale goes from 1 (very pleasant) to 6 (very unpleasant).

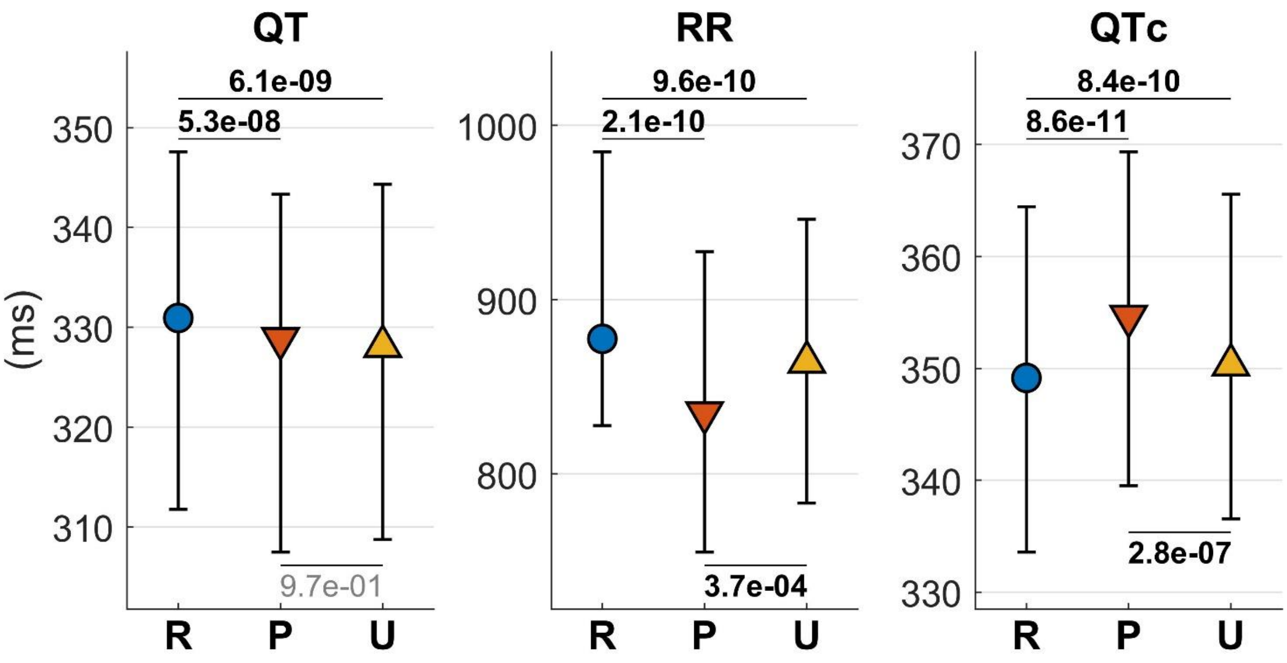

Supplementary Figure 2. Analysis of lead II. QT, RR and QTc (QT corrected for heart rate) during listening to pleasant music (P), unpleasant music (U) and rest (R). Markers represent the median values and bars span from the first to the third quartile. P-values measuring pair-wise differences are reported in bold if significant and in light grey if not significant. For comparison with results from lead V4, please see Figure 3.

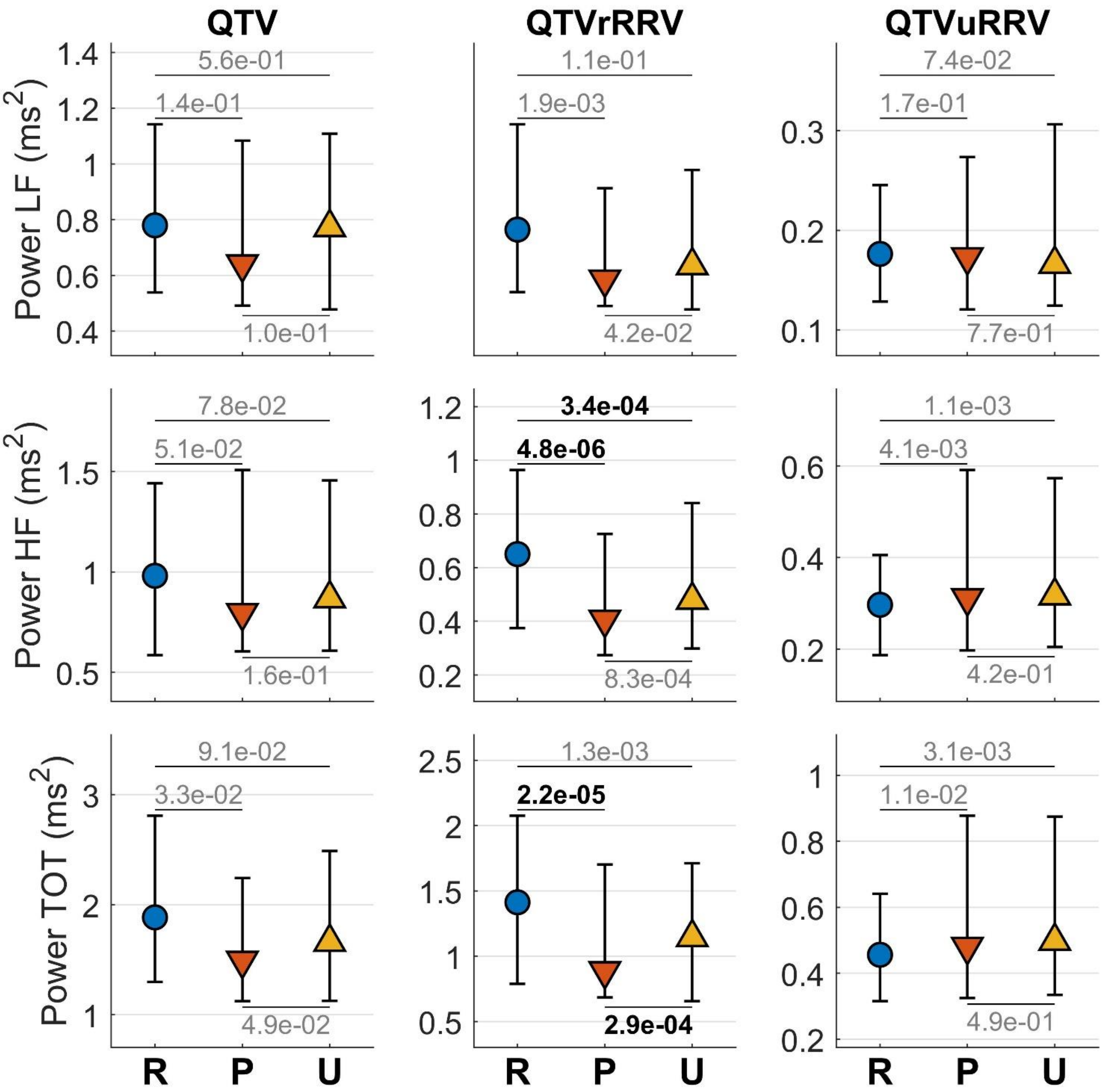

Supplementary Figure 3: Analysis of lead II. QTV and QTV related and unrelated to RRV during listening to pleasant music (P), unpleasant music (U) and rest (R). From left to right: QTV, QTV related to RRV (QTVrRRV) and QTV unrelated to RRV (QTVrRRV). From top to bottom: Mean power of LF, HF and total spectral band. Markers represent the median values and bars span from the first to the third quartile. P-values measuring pair-wise differences are reported in bold if significant and in light grey if not significant. For comparison with results from lead V4, please see Figure 6.
